# Early Enteral Feeding Restores Neurofilament Light Chain Serum Levels in Preterm Newborns

**DOI:** 10.2174/1570159X23666240920165612

**Published:** 2024-11-07

**Authors:** Maria Di Chiara, Gianluca Terrin, Marco Fiore, Maria Chiara De Nardo, Gianluigi Laccetta, Flavia Gloria, Antonio Minni, Christian Barbato, Carla Petrella

**Affiliations:** 1 Department of Mother and Child Health, Policlinico Umberto I, Sapienza University of Rome, Rome, Italy;; 2 Institute of Biochemistry and Cell Biology (IBBC) of the National Research Council (CNR), Rome, Italy;; 3 Department of Sense Organs DOS, Sapienza University of Rome, Rome, Italy;; 4 Division of Otolaryngology-Head and Neck Surgery, San Camillo de Lellis Hospital, ASL Rieti-Sapienza University, Rieti, Italy

**Keywords:** Pre-terms, early enteral feeding, nutrition, neurofilament light chain, neurodevelopment, biomarker

## Abstract

**Background:**

Positive effects of early nutritional strategies on neurological outcomes have been observed when nutrients were administered by the enteral route, especially during the first week of life. Evidence reports that serum neurofilament light chain (NfL), a structural protein of neurons, is a specific and reliable biomarker of neuronal damage.

**Objective:**

The present study aimed to investigate the effect of early enteral nutrition (EN) in minimizing neuroaxonal damage and assessing NfL serum levels in preterm neonates.

**Methods:**

Fifty-four preterm neonates without severe brain impairment and 20 full-term babies as controls were enrolled from the Neonatal Intensive Care Unit at the Policlinico Umberto I in Rome. We performed blood sampling at birth (day of life 0 - DoL 0) in 20 full-term newborns and in 19 pre-term infants. Furthermore, we executed blood sampling at DoL 28 in other 22 pre-term newborns who received early enteral nutrition (EN) within the third DoL (Early-EN) and in 13 other pre-term newborns who received EN after the third DoL (Late-EN).

**Results:**

Serum levels of NfL were higher in preterm babies when compared to full-term neonates, at DoL 0 (48.81 ± 9.4 *vs*. 11.67 ± 1.4 pg/ml; *p* = 0.007). Interestingly, at DoL 28, serum NfL was significantly decreased in the Early-EN newborns compared to the Late-EN groups (15.22 ± 2.0 *vs*. 50.05 ± 17.9 pg/ml; *p* = 0.03).

**Conclusion:**

It was shown that early enteral feeding, within the first week of life, could be a useful tool for limiting neurological impairment in pre-term neonates by restoring NfL.

## 1 INTRODUCTION

The objective of artificial nutrition in preterm infants is to optimize growth and ensure a growth rate similar to that in utero [1]. In premature newborns, conditions such as reduced supplies of carbohydrates and lipids, high energy expenditure linked to the presence of metabolically very active tissues, increased water dispersion, and immaturity of the digestive system limit and sometimes prevent normal functioning oral feeding [1, 2].

These conditions expose the newborn to a significant nutritional risk even in a very short time. Recommendations in clinical practice suggest starting artificial nutrition early in life to avoid catabolism as a consequence of fasting rather than of the preterm state [3, 4].

The choice of the feeding route is still the object of discussion [5-7]. Independently from the parenteral nutrition (PN) or enteral nutrition (EN) route, it’s widely proven that the introduction of minimal enteral feeding early in life, is a strategy to stimulate the development of the immature gastrointestinal tract of the preterm infant, resulting in clinical benefits including improved tolerance to EN, increased postnatal growth, and reduction of systemic sepsis [8-10]. Although in some cases (such as in very low body weight preterm), PN is the only one indicated [11], the introduction of EN, as soon as the conditions of the newborn are stable, seems to be particularly effective in reducing metabolic intolerance problems related to PN and in promoting adequate neurological development in the long term, through the activation of the gut-brain axis [12]. Recently, it was demonstrated that EN, given early in life, may reduce PN side effects [9]. Our previous study, including premature newborns, demonstrated that Nerve Growth Factor (NGF) and Brain-Derived Neurotrophic Factor (BDNF) serum levels positively correlated with enteral protein and energy intake administered in the first week of life [13]. Thus, the study of mechanisms by which EN has an impact on neurological outcomes may provide a tool to optimize nutritional strategies to limit neuronal impairment. In the past decades, several serum markers have been proposed to detect the changes and the pathophysiological reactions to a neuronal injury. These molecules have been identified as a potential biomarker, reflecting processes of either glial and/or axonal injury [14]. In particular, in response to an axonal injury, the release of neurofilament light chain (NfL), a protein constituting part of the neuronal cytoskeleton, sharply increases in peripheral sites [15]. Indeed, recent pieces of evidence have reported that NfL is a specific and reliable biomarker of neuronal damage in preterm newborns [16, 17]. Moreover, NfL measured during the first weeks of life predicted motor outcomes in preterm infants with peri/intraventricular hemorrhage [18]. To date, to the best of our knowledge, no studies investigated the relationship between circulant NfL and the postnatal nutritional approach, in preterm infants. Thus, this study aimed to assess whether or not early enteral administration of nutrients could influence NfL serum levels, becoming a biomarker for the short-term neuroprotective effect of the nutritional strategy. We predicted that the early EN strategy could counteract NfL elevation in preterm infants.

## 2 MATERIALS AND METHODS

### 2.1 Neonatal Groups and Study Design

The present study involved 74 newborns recruited at the Neonatal Intensive Care Unit at the Sapienza University Hospital Policlinico Umberto I in Rome between the years 2020 and 2022. To minimize the number of blood withdrawals in newborns, we performed the venous blood sampling at birth (day of life 0 - DoL 0) in 20 full-term (*Full-Term*) newborns and in 19 pre-term (*Pre-Term*) infants with gestational age (GA) < 32 weeks and/or body birth weight (BW) < 1500 g. Furthermore, we performed the venous blood sampling at DoL 28 in 22 other pre-term newborns who received early enteral nutrition (EN) within the third DoL *(Early-EN)* and in other 13 pre-term newborns who received EN after the third DoL (*Late-EN*). The 20 *full-term* newborns were used as a control group. Fig. (**1**) represents the study design. The main exclusion criteria to avoid any bias in the selection for the newborn enrolment (including controls) included: 1) Newborns with congenital intestinal or extra-intestinal malformations, intraventricular hemorrhage (IVH) stage ≥ 3, congenital infections, inborn errors of metabolism, and genetic syndromes. 2) Hospital transfer within DoL 28 (except for *Full Term* controls who were discharged home at 48-72 hours of life) or death, critical conditions in the first 48 hours after birth (*i.e*., patients who were not expected to survive), incomplete clinical data, and absence of written informed consent by parents or baby’s legal guardian.

### 2.2 Nutritional Protocol

Enteral nutrition (EN) was started as soon as possible after birth in a newborn with stable hemodynamic status. Minimal enteral feeding was initiated in all recruited patients within 24-48 hours of birth with a dose of 10-20 ml/kg/day. The amount was increased by 20-30 ml/kg each day if EN was tolerated. Unfortified breast milk, if available, was administered fresh. If breast milk was not available or not sufficient, a specific formula for preterm infants was used. When signs or symptoms of food intolerance such as vomiting, severe abdominal distension associated with ileus with visible intestinal loops, blood in the stool, or systemic disorders (*e.g*., apnea, bradycardia, inadequate perfusion, and hemodynamic instabilities) were observed, the EN was withheld for at least 24 hours. The macronutrient content of the formula and parenteral solutions was calculated based on the manufacturers' published records. Parenteral administration of nutrients (PN) was performed from birth to maintain an adequate intake of fluids, electrolytes, and nutrients until exclusive enteral feeding was achieved (120 ml/kg/day). Overall fluid intake administered by enteral and PN was started at 80 ml/kg/day and slowly increased by 10–20 ml/kg/day until reaching 150 ml/kg/day. In PN, we administered 2.5 g/kg/day of amino acids (TrophAmine^®^ 6% Braun Medical Inc. Irvine, USA) in the first DoL and then increased the amino acid intake up to 3.2 g/kg/day, with 25 kcal per 1 g of amino acids. Glucose intake (10% dextrose injection, Fresenius Kabi, USA) was started at 6 g/kg/day and increased to 13 g/kg/day. Lipid intake (Smoflipid^®^, Fresenius Kabi, USA) was started at 1 g/kg/day and increased to 3.5 g/kg/day. Preterm breast milk was assumed to contain 65 kcal/100 ml (1.5 g protein/100 ml, 3.5 g fat/100 ml, 6.9 g carbohydrates/100 ml). The macronutrient content of the formula (Pre-Nidina Nestlè^®^: proteins 2.9 g/dl, lipids 4.0 g/dl, carbohydrates 8.1 g/dl) was calculated based on the labels published by the manufacturer. Total energy intake was calculated based on the cumulative amount of PN and EN in kcal/kg in the first 7 DoL. The target dose refers to EN plus PN; therefore, we adjusted PN intake based on the amount of EN tolerated.

### 2.3 Data Collection

Prenatal, perinatal, and postnatal information were prospectively collected from all patients recruited. Gestational age (GA), body weight (BW), gender, Appearance (skin color), Pulse, Grimace, Activity, Respiratory effort (APGAR) score at 1^st^ and 5^th^ min after birth, mother’s age, starting EN, PN duration, and hospitalization were recorded. Diagnosis of the major morbidities associated with prematurity, such as necrotizing enterocolitis (NEC, Bell stage ≥ 2), bronchopulmonary dysplasia (BPD), intraventricular hemorrhage (IVH), periventricular leukomalacia (PVL), retinopathy of prematurity (ROP), and sepsis proven by positive cultures was performed according to the standard criteria and recorded in the reporting form. The total number of co-morbidities was compared between the enrolled groups.

### 2.4 NfL Serum Measurement

Specimens of blood (0.5 ml) were obtained from preterm newborns at DoL 0 and DoL 28; a sample of blood from full-term control babies at DoL 0 was also collected. In both cases, specimens at DoL 0 were collected from umbilical cord blood. Biological samples were contained in 500 μl-serum microvettes, which were centrifuged at 3000 rpm for 15 minutes to separate the serum. The serum was then stored at -20°C pending analysis. NfL was measured using a sandwich enzyme-linked immunosorbent assay (ELISA) kit (Cat. No. 20-8002, UmanDiagnostics, Sweden) according to the protocols provided by the manufacturer. Serum samples were diluted 4-fold and tested in duplicate. The colorimetric reaction product was measured at 450 nm using a microplate reader (Dynatech MR 5000, PBI International, USA). Data were represented in pg/ml.

### 2.5 Statistical Investigation

All data were evaluated using a two-way analysis of variance (ANOVA) to take into account the sex effect. However, in the absence of a sex effect, the data were analyzed using a one-way ANOVA. Post hoc comparisons were analyzed using Tukey’s HSD test. The Spearman nonparametric test was utilized to determine the correlations between parameters in the groups. Data normal distribution was also analyzed. All data were analyzed by JASP (the University of Amsterdam, version 0.16 for the Mac).

### 2.6 Ethics

The present study was conducted in conformity with the World Medical Association Declaration of Helsinki for medical research involving human subjects. This investigation reports a part of the results of the study protocol n° 5089, which was approved by the Ethics Committee of Policlinico Umberto I Hospital, Sapienza University of Rome. Written informed consent was obtained from parents, and anonymized data were collected in an appropriate form.

## 3 RESULTS

### 3.1 NfL Serum Measurement

ANOVA data showed that circulating levels of NfL were influenced by the GA (*Full-Term vs. Pre-Term*) and by nutritional intervention (early and late enteral feeding) (F(3,70), 6.547; *p <* 0.001). As shown in Fig. (**2**), NfL significantly augmented in the serum of pre-term infants in comparison with full-term babies at birth (DoL 0) (*p <* 0.01). Moreover, different starting times for EN strongly impacted the NfL levels in pre-term newborns after 28 days (DoL 28) (*p <* 0.05). In particular, in Early-EN newborns, the administration of enteral feeding within the first three days of life restored the NfL levels with values comparable to those of full-term infants, whilst in Late-EN patients, the late starting time of EN left NfL serum unchanged, in comparison with those of pre-terms at the birth.

### 3.2 Perinatal, Postnatal, and Clinical Data

Table **1** shows the main perinatal and clinical characteristics of the recruited newborns. Data regarding full-term and pre-term newborns were used to evaluate the relationship between GA and serum NfL levels at DoL 0. Data about early-EN and late-EN newborns were analyzed to assess the relationship between the timing of the introduction of EN and short-term potential neuronal implication (DoL 28) through NfL measurement.

Full-term and pre-term infants demonstrated, as expected, different GA and body weight at DoL 0 (*p <* 0.001). Concerning early-EN and late-EN pre-term newborn body weight, no differences were found in comparison to pre-term infants at DoL 0. However, a significant dissimilarity in body weight between early-EN and late-EN newborns was observed both on DoL 0 (*p* = 0.02) and DoL 28 (*p* = 0.03), with body weight of late-EN infants lower than of early-EN newborns. As predicted, sex differences in body weight were found at DoL 28. However, weight increase expressed as a percentage in both Early-EN and Late-EN newborns were similar, revealing that, independently from the nutrition protocol, all newborns had comparable growth.

In Early-EN and Late-EN infants, no differences were disclosed for the GA, the APGAR score (1’ and 5’), and the number of morbidities, strongly supporting the comparability of the recruited infants in terms of growth development (gestational age) and global health condition at birth (APGAR and morbidities).

Finally, significant reductions in the duration of PN and hospitalization in early-EN newborns were observed when compared to pre-term infants receiving late enteral feeding (Late-EN).

### 3.3 Correlation Analysis

Spearman’s analyses (Table **2**) revealed an inverse correlation between NfL serum levels and APGAR 1’ score in the Late-EN group, indicating that, inside this group, low score values (worse health conditions) matched with high NfL levels. Intriguingly, in all infants (both early-EN and late-EN) on DoL 28, a direct correlation between NfL and PN duration was found, evidencing that the high serum NfL corresponded to long-lasting PN.

## 4 DISCUSSION

The study showed, for the first time, to the best of our knowledge, the short-term relation (DoL 28) between early EN and serum NfL, displaying a potential biomarker of the well-known positive effect of enteral nutrition in preterm infants. In particular, we demonstrated that premature newborns starting EN within three days of life (mean starting day in Early-EN group: 0.95 ± 0.12) reduced NfL serum levels, after DoL 28, with values comparable to those of full-term infants at birth; on the contrary, pre-terms receiving later EN (mean starting day in Late-EN group: 7.0 ± 1.5) showed NfL serum concentrations comparable to those of pre-terms at birth. Interestingly, low NfL levels in early-EN infants were associated with shorter PN duration and hospitalization compared to the late-EN group, which exhibited high NfL. Similarities in gestational age, APGAR scores, number of comorbidities, and growth rate during the period considered in our study (DoL 28) between the two enrolled groups (Early- and Late-EN) strongly supported the role of early EN in the protection of neuronal integrity, measured through NfL circulating levels. Finally, the lack of severe brain impairments (exclusion criteria, potentially confounding factors in the elevation of serum NfL) in all the pre-terms analyzed strongly supports NfL as a short-term reliable biomarker to validate the neuroprotective role of early enteral nutrition.

Pre-term birth is accompanied by an immaturity of many organs and systems, with a consequent difficulty in dealing with the extra-uterine environment, which becomes more evident with decreasing gestational age [19]. In severe cases, where prematurity is associated with neurological insults, deficits in cognitive abilities and motor skills have been discovered [20]. The nervous system of the newborn is characterized by extreme plasticity until its maturation around two years of life: this makes it, on the one hand, very vulnerable and, on the other, extremely responsive to sensory, environmental, and relational stimuli [21]. Thus, the establishment of early post-natal interventions is aimed at modifying developmental trajectories by directing them toward a positive outcome.

Regarding postnatal neuroprotective strategies, nutrition plays an emerging role [22-24].

Although current nutritional practices are not able to mimic the exponential accumulation of nutrients that normally occurs during the third trimester of pregnancy, necessary for optimal postnatal growth and development [24], growing scientific evidence supports the idea that the routes of administration of nutrients could exert a long-term neurodevelopmental effect in premature infants [25, 26].

A systematic review on the effect of enteral and parenteral energy intakes on neurodevelopment and cerebral growth in pre-term infants disclosed that energy intake, mainly by EN, had a positive impact on brain development and suggests a more cautious approach to enhanced nutritional strategies by the parenteral route [27]. Our previous study, including that of premature newborns, demonstrated that NGF and BDNF serum levels at DoL 28 positively correlated with enteral protein and energy intakes administered in the first week of life [13]. An increase in the neurotrophin’s circulant levels, however, could not be the only mechanism involved in the relationship between EN and neurodevelopment in preterm-born babies.

Enteral feeding can exert a trophic effect on the intestine, either through the direct action of growth factors present, for example, in fresh breast milk, or indirectly, following the release of intestinal hormones. Numerous studies have shown that plasma concentrations of enteroglucagon, motilin, neurotensin, and gastro-inhibitory peptide were significantly higher in enterally-fed infants compared to those receiving parenteral nutrition alone [28]. Early enteral feeding, by stimulating the secretion of intestinal peptides, could, therefore, play a crucial role in the physiological adaptation to extrauterine nutrition. Furthermore, although intestinal motor activity in preterm infants is “immature” when compared to that of full-term infants and older children, its maturation may be influenced by the onset of enteral nutrition. Infants receiving early enteral nutrition have been shown to exhibit greater migratory motor activity, responsible for aboral propulsion of nutrients, compared to infants receiving parenteral nutrition alone [29, 30].

Neurofilaments are structural proteins of the neuronal cells of the central and peripheral nervous system that are essential for maintaining the correct structure of nerve cells and are necessary for the effective conduction of electrical impulses along the axons. The light chain is a component of the neurofilament that is released following axonal damage and/or neuronal degeneration [15].

By focusing on NfL as a biomarker for neurodegenerative decline, most of the scientific literature investigated its potential role in the onset, progression, and prognosis of most neurodegenerative diseases (Alzheimer’s disease, Parkinson’s disease, Multiple Sclerosis) [31-34].

However, there is a dearth of adequate evidence centering on the role of NfL in preterm neonates. Retrospective studies were focused mainly on the comparison of serum NfL between pre-terms affected by neurological disorders and full-term neonates, disclosing higher serum NfL levels in pre-terms compared to those of neonates at term. Furthermore, NfL levels were negatively correlated with both GA and BW and NfL levels increased along with newborns’ days of life in preterm neonates [17]. In a prospective observational study including preterm newborns with moderate to severe peri/intraventricular hemorrhage, it has been demonstrated that serum NfL levels were an independent predictor of motor outcome, implying that this protein could be used to identify neonates at high risk of neurodevelopment disabilities. Sjobom *et al*., in a longitudinal retrospective study, found a positive correlation between NfL serum levels, measured between the second and the fourth week of life, and unfavorable neurodevelopment outcomes, assessed by the Bayley Scales of Infant Development, at 2 years of age, in preterm neonates [35]. Few studies have already demonstrated the role of NfL in the diagnosis and prognosis of hypoxic-ischemic encephalopathy and the prediction of neurodevelopmental outcomes in preterm-born infants [36]. Toorell *et al*. demonstrated that cord blood NfL levels were higher in term newborns with intrapartum asphyxia and correlated with the severity of the insult based on the Apgar score at 5 minutes, arterial cord blood pH, and Sarnat clinical staging of hypoxic-ischemic encephalopathy [36]. In the investigation by Shah *et al*., [16, 37] term newborns with moderate-severe hypoxic-ischemic encephalopathy treated with therapeutic hypothermia were studied. In this study, NfL concentrations were measured on plasma samples when the infant reached the target temperature, before commencing rewarming, and after completing rewarming [37]. Newborns with MRI were predictive of an unfavorable outcome and had significantly higher levels of NfL compared to those with favorable outcomes at all three-time points [37].

The strength of our research is emphasized by the stringent exclusion criteria, which, although limiting the sample size, allowed us to highlight how the variations in NfL, both at birth and after 4 weeks of life, were not due to brain impairments.

NfL, therefore, can be an indicator of neuronal damage even in the absence of critical central events and can be modulated in response to a different nutritional approach. Therefore, NfL could be considered a crucial biomarker of the effectiveness of the nutritional strategy in premature infants.

The findings of the present investigation, however, should be interpreted considering some limitations. For ethical reasons, it was not possible to design a randomized controlled trial evaluating the nutritional support of critically ill neonates. Furthermore, randomization in these patients puts the study at high risk of losing a considerable proportion of neonates due to lack of equipoise. At the same time, individualized corrections of PN solutions are the milestone of our policy on PN to avoid the deleterious consequences of complications related to the fourth administration of these solutions [16, 37-40]. Thus, physicians must know details about enteral and parenteral administration of nutrients in enrolled newborns, which makes double-blind studies impracticable in our reality. However, no changes in the care policies during the study period (except for the timing of the introduction of EN) occurred; even the preterm formula used for EN (in case maternal milk was lacking) remained the same over time. The small sample size, due to the stringent exclusion criteria in the patient enrolment, is also a critical point. Finally, we did not analyze data on long-term neurodevelopment. However, it should be noted that our study focused on the relationship between enteral nutrition early in life and NfL levels in a short-term period. Further studies are deemed to establish if this relation is confirmed when investigating long-term neurodevelopment. It would be interesting to collect serum NfL levels at DoL 0 and DoL 28 from the same newborns, following them over time to verify the long-term protective effect of the early enteral nutrition strategy on neurological development and its correlation with the new potential biomarker.

## CONCLUSION

All together, these results suggest that the administration of nutrition through the enteral route early in life may be worthwhile for limiting neurological impairments in neonates at elevated risk of cerebral damage. For the first time, we propose serum NfL as a promising diagnostic and prognostic approach for monitoring the beneficial effects of early enteral nutrition in the neurodevelopment of premature neonates. This study encourages further research on the clinical role of NFL to translate into the nutritional and medical management of preterm newborns at risk of brain damage.

## AUTHORS’ CONTRIBUTIONS

It is hereby acknowledged that all authors have accepted responsibility for the manuscript's content and consented to its submission. They have meticulously reviewed all results and unanimously approved the final version of the manuscript.

## Figures and Tables

**Fig. (1):**
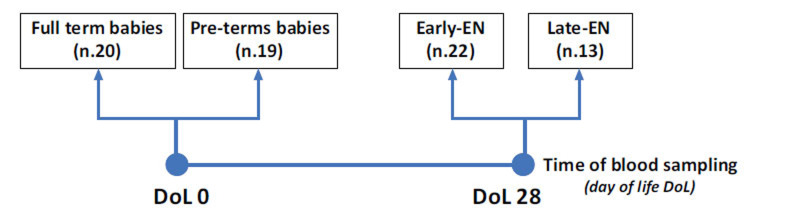
Schematic representation of the study design. The group of full-term babies and the group of pre-term newborns were blood sampled at birth (DoL 0). Early-EN (who received enteral nutrition within the third DoL) and late-EN (who received enteral nutrition after the third DoL) groups were blood samples at DoL 28.

**Fig. (2):**
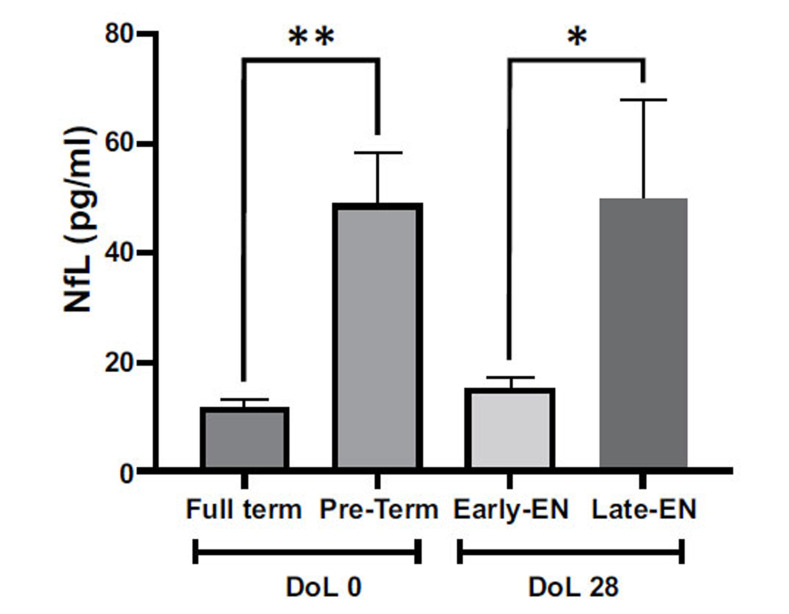
Serum NfL in the recruited individuals. Data are presented as mean ± standard error means (SEM). The asterisks (*p < 0.05: **p < 0.01) indicate post-hoc differences between groups. DoL (day of life).

**Table 1:** Summary of the main characteristics of the newborns recruited for the study.

**Blood Withdrawal at DoL 0**					
**-**		**Full Term ** **(n. 20; M10/F10)**	**Pre-term** **(n.19; M9/F10)**	***P***	**(df), F**
GA (weeks)	tot	38.6 ± 0.1	29.5 ± 0.8	< 0.001	(1,37), 309.4
	M	38.7 ± 0.2	31.1 ± 1.1	*P*_(treatment)_ < 0.001 *P*_(gender)_ 0.03	(1,35) F_(treatment)_ 371.357 F_(gender)_ 5.148
	F	38.6 ± 0.2	28.0 ± 0.9		
BW DoL 0 (g)	tot	3383.3 ± 89.2	1272 ± 106.4	< 0.001	(1,37), 232.8
	M	3332.2 ± 102.4	1527.3 ± 128.2	*P*_(treatment)_ < 0.001 *P*_(gender)_ 0.151	(1,35) F_(treatment)_ 261.234 F_(gender)_ 2.160
	F	3434.5 ± 150.3	1043.5 ± 132.4		
**Blood Withdrawal at DoL 28**					
**-**		**Early-EN** **(n. 22; M10/F12)**	**Late-EN** **(n. 13; M4/F9)**	***p*-value**	**(df), F**
Start EN, DoL (days)	tot	0.95 ± 0.12	7.0 ± 1.5	<0.001	(1,33), 28.405
	M	1.10 ± 0.57	10.5 ± 3.9	*P*_(treatment)_ <0.001 *P*_(gender)_ 0.02	(1,31), F_(treatment)_ 40.530 F_(gender)_ 5.848
	F	0.83 ± 0.17	5.44 ± 1.1		
GA (days)	tot	216.7 ± 2.0	208.7 ± 4.7	0.08	(1,33), 3.301
	M	217.6 ± 2.1	219.0 ± 9.6	*P*_(treatment)_ 0.254 *P*_(gender)_ 0.07	(1,31), F_(treatment)_ 1.349 F_(gender)_ 3.333
	F	216.0 ± 3.3	204.1 ± 5.9		
APGAR 1’	tot	6.54 ± 0.47	5.23 ± 0.64	0.103	(1,33), 2.812
	M	5.90 ± 0.9	6.75 ± 0.9	*P*_(treatment)_ 0.301 *P*_(gender)_ 0.531	(1,31), F_(treatment)_ 1.107 F_(gender)_ 0.402
	F	7.08 ± 0.4	4.56 ± 0.7		
APGAR 5’	tot	8.09 ± 0.3	7.61 ± 0.4	0.408	(1,33), 0.702
	M	7.30 ± 0.7	8.50 ± 0.3	*P*_(treatment)_ 0.771 *P*_(gender)_ 0.878	(1,31), F_(treatment)_ 0.086 F_(gender)_ 0.024
	F	8.70 ± 0.1	7.22 ± 0.6		
BW DoL 0 (g)	tot	1459.4 ± 53.4	1204.6 ± 100	0.019	(1,33), 6.079
	M	1485.0 ± 96.4	1323.7 ± 168.2	*P*_(treatment)_ 0.05 *P*_(gender)_ 0.330	(1,31), F_(treatment)_ 4.096 F_(gender)_ 0.981
	F	1438.0 ± 59.8	1151 ± 125.9		
BW DoL 28 (g)	tot	1892.3 ± 71.6	1573.9 ± 83.6	0.008	(1,33), 7.906
	M	1943.0 ± 109.5	1739.0 ± 133.6	*P*_(treatment)_ 0.03 *P*_(gender)_ 0.175	(1,31), F_(treatment)_ 5.363 F_(gender)_ 1.923
	F	1850.0 ± 96.9	1550.6 ± 99.9		
ΛBW (%)	tot	18.9 ± 2.0	18.4 ± 3.0	0.890	(1,33), 0.019
	M	20.9 ± 2.8	22.2 ± 4.8	*P*_(treatment)_ 0.918 *P*_(gender)_ 0.228	(1,31), F_(treatment)_ 0.011 F_(gender)_ 1.514
	F	17.2 ± 2.9	16.8 ± 3.9		
PN duration (days)	tot	11.7 ± 1.8	26.5 ± 5.9	0.006	(1,33), 8.638
	M	8.70 ± 2.1	19.5 ± 7.8	*P*_(treatment)_ 0.019 *P*_(gender)_ 0.152	(1,31), F_(treatment)_ 6.115 F_(gender)_ 2.161
	F	14.2 ± 2.6	29.7 ± 7.5		
Hospitalization (days)	tot	50.1 ± 2.6	67.6 ± 7.7	0.014	(1,33), 6.705
	M	47.9 ± 4.4	54.2 ± 10.0	*P*_(treatment)_ 0.05 *P*_(gender)_ 0.107	(1,31), F_(treatment)_ 3.972 F_(gender)_ 2.758
	F	51.9 ± 3.2	73.6 ± 9.8		
Morbidities (n.)	tot	0.64 ± 0.2	1.07 ± 0.3	0.210	(1,33), 1.635
	M	0.40 ± 0.2	0.50 ± 0.3	*P*_(treatment)_ 0.407 *P*_(gender)_ 0.09	(1,31), F_(treatment)_ 0.705 F_(gender)_ 3.143
	F	0.83 ± 0.4	1.3 ± 0.3		
Mother’s age (years)	tot	34.0 ± 1.4	33.2 ± 1.3	0.697	(1,33), 0.154
	M	32.3 ± 2.2	30.5 ± 3.0	*P*_(treatment)_ 0.513 *P*_(gender)_ 0.108	(1,31), F_(treatment)_ 0.437 F_(gender)_ 2.736
	F	35.5 ± 1.8	34.4 ± 1.3		

**Note:** *p* values < 0.05 are considered significant.

**Table 2:** Spearman’s correlation matrix between NfL (pg/ml) serum levels at DoL 28 and the variable indicated.

**Pre-Term Early-EN Group (n = 22)**									
**Nfl *vs.***	**ΔBW%**	**n. Morbidity**	**Hospitalization (Days)**	**GA (Days)**	**PN Duration (Days)**	**APGAR 1'**	**APGAR 5'**	**pH**	
Spearman r	-0.079	0.191	0.132	0.192	0.230	-0.009	-0.070	0.243	
*P* (two-tailed)	0.724	0.394	0.557	0.391	0.302	0.964	0.756	0.287	
*P* value summary	ns	ns	ns	ns	ns	ns	ns	ns	
**Pre-Term Late-EN Group (n = 13)**									
**Nfl *vs.***	**ΔBW%**	**n. Morbidity**	**Hospitalization (Days)**	**GA (Days)**	**PN Duration (days)**	**APGAR 1'**	**APGAR 5'**		**pH**
Spearman r	0.243	0.262	0.179	0.243	0.484	-0.583	-0.517		0.452
*P* (two-tailed)	0.415	0.382	0.554	0.419	0.095	0.039	0.075		0.121
*P* value summary	ns	ns	ns	ns	ns	*	ns		ns
**Total Individuals (Pre-Term Early and Pre-Term Late-EN) (n = 35)**									
**Nfl *vs.***	**ΔBW%**	**n. Morbidity**	**Hospitalization (Days)**	**GA (Days)**	**PN Duration (Days)**	**APGAR 1'**	**APGAR 5'**		**pH**
Spearman r	0.017	0.264	0.224	0.131	0.436	-0.328	-0.314		0.320
*P* (two-tailed)	0.922	0.124	0.194	0.450	0.008	0.054	0.065		0.064
*P* value summary	ns	ns	ns	ns	**	ns	ns		ns

**Note:** Asterisks indicate significant values (*p < 0.05; **p < 0.01).

## Data Availability

The datasets analyzed during the current study are available from the corresponding authors (G.T and C.P) upon reasonable request.

## LIST OF ABBREVIATIONS

BW: Body Weight
DoL: Day of Life
EN: Enteral Nutrition
GA: Gestational Age
NFL: Neurofilament Light Chain
PN: Parenteral Nutrition
